# Theoretical and experimental investigation of the excellent p–n control in yttrium aluminoborides

**DOI:** 10.1088/1468-6996/15/3/035012

**Published:** 2014-06-24

**Authors:** Ryoji Sahara, Takao Mori, Satofumi Maruyama, Yuzuru Miyazaki, Kei Hayashi, Tsuyoshi Kajitani

**Affiliations:** 1 National Institute for Materials Science, Research Center for Strategic Materials, 1-2-1 Sengen, Tsukuba, Ibaraki 305-0035, Japan; 2 National Institute for Materials Science, International Center for Materials Nanoarchitectonics, 1-1 Namiki, Tsukuba, Ibaraki 305-0044, Japan; 3 University of Tsukuba, 1-1-1 Tennodai, Tsukuba, Ibaraki 305-8577, Japan; 4 Department of Applied Physics, Graduate School of Engineering, Tohoku University, 6-6-05 Aoba, Aramaki, Aoba, Sendai, Miyagi 980-8579, Japan

**Keywords:** first principles calculation, thermoelectric materials, yttrium aluminoborides

## Abstract

First-principles calculations were carried out to elucidate the excellent control of p–n characteristics recently reported for yttrium aluminoborides Y_x_Al_y_B_14_ 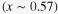 with different occupancies of Al sites 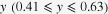. Such control of the occupancy of metal sites in borides is unusual. Calculations based on detailed x-ray diffraction data reveal a stable configuration of the atomic sites, indicating that such variation in occupancy is possible. A shift from positive through zero to negative values of the Seebeck coefficient is also clearly illustrated by determining the density of states for different configurations.

## Introduction

The usefulness of direct conversion of waste heat into electricity is a strong incentive for developing viable thermoelectric (TE) materials, and such development is actively conducted across the globe by using state-of-the-art nanotechnology and nanomaterials [1, 2]. Materials that can function at high temperature are particularly sought after. In this regard, boron cluster compounds are attractive materials due to their stability and high melting points above 2200 K. Many novel compounds of this type with interesting structures have been discovered in recent years [3]. Furthermore, such compounds have been found to possess low thermal conductivity, *κ*, even for single crystals, which is an inherent advantage for TE application [4–8]. The main constituent element, boron, is also relatively abundant and nontoxic, whereas traditional TE materials often include expensive and/or toxic elements, such as bismuth, tellurium, lead, and silver.

Boron carbide is a good p-type high-temperature TE compound, and one of the few commercialized TE materials [9, 10]. However, TE modules require p-type and n-type materials with matching structure, and the lack of a suitable matching n-type counterpart for boron carbide has been a longstanding problem since icosahedral boron compounds have been found to be predominantly p-type. Slack first showed the n-type characteristics of vanadium-doped *β*-boron [11, 12], and extensive tests have been conducted for various dopants [13–15]. However, all such n-type borides undergo metallization, whereby the Seebeck coefficient decreases with increasing temperature, in contrast to the temperature dependence of boron carbide. Recently, a series of rare earth borocarbonitrides, *R*B_17_ CN and *R*B_22_C_2_ N (*R* = rare earth), which are in homologous structural relationship with boron carbide, were considered to be the long-awaited intrinsically n-type counterparts of boron carbide [16, 17]. However, these compounds are hard to densify, and this problem is being investigated [18, 19]. *R*B_44_Si_2_ is another novel boride with Seebeck coefficient, *α*, greater than 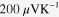 at high temperature, and unlike most compounds, the dimensionless figure of merit, *ZT*, shows a steep increase at above 1000 K [20]. Boron sulfide B_6_S_1−*x*_ also has a large Seebeck coefficient, but with potentially much lower processing temperature in comparison with typical borides [21].

In a recent striking development, it was discovered that yttrium aluminoborides Y_*x*_Al_*y*_B_14_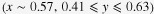 allow for excellent variation of 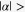 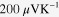, showing p-type or n-type characteristics depending on the *y* occupancy of Al sites [22]. Since the difficulty of controlling the p–n characteristics of materials has traditionally been one of the major obstacles to developing applicable TE materials, the discovery of a method for such control in a group of compounds with the same basic crystal structure (therefore ensuring close structural match) and no need for doping of foreign elements (therefore eliminating migration problems) is extremely valuable. This discovery is furthermore surprising since in metal borides the possible variation in the occupancy of metal sites has been found to be rather small, for example, in *M*B_6_, *M*B_12_, *M*B_25_, *M*B_66_ (*M* = metal), the recently discovered borides mentioned above, and even yttrium aluminoborides such as *R*AlB_4_ [23] and the previously reported *R*AlB_14_ (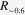
Al_∼0.7_B_14_) itself [24, 25].

**Figure 1:**
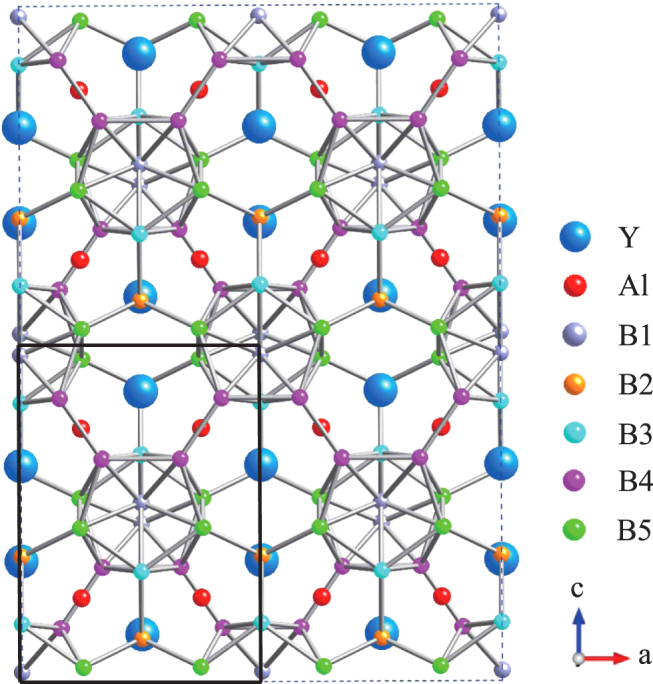
Crystal structure of Y_*x*_Al_*y*_B_14_.

Against this background, to elucidate this surprising behavior we carried out a theoretical investigation on 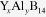 based on crystallographic parameters determined from detailed x-ray diffraction (XRD) Rietveld analysis of yttrium aluminoborides. Figure 1 shows the schematic structure of YAlB_14_, where the *a–c* plane of the compound is viewed directly from above. The black square in the lower left corner of the figure corresponds to one unit cell of the compound. The compound has four formula units in one orthorhombic cell with space group *Imma*. There are crystallographically independent boron atoms (B1, B3, B4, and B5), which form B_12_ icosahedra, and bridging boron atoms (B2), which are located between the B_12_ atoms.

Here we consider a series of Y_*x*_Al_*y*_B_14_ compounds with varying *x* and *y* and study their electronic properties through total energy calculations in order to understand the effects of metal concentrations on the control of p–n characteristics. In section 2, we discuss our experimental and calculation procedure, and the results are given in section 3. A summary is given in section 4.

## Experimental and calculation methods

The crystallographic parameters for the calculations were determined from Rietveld refinement of actual Y_*x*_Al_*y*_B_14_ 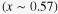 samples synthesized as described previously [22]. The starting materials YB_4_ (99.9%, Japan New Metals), B (99%, New Metals and Chemicals), and excess Al which served as flux (99%, Wako Pure Chemical Industries) were mixed with nominal composition of Y_0.56_Al_*y*_B_14_ 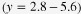, and a modified Al flux method was used. In addition to the samples in [22], a sample with *y* = 0.39 was also newly synthesized because in the previous study there was only one sample with a composition in the positive Seebeck coefficient range. The detailed synthesis conditions of the *y* = 0.39 sample is as follows. Starting materials of 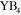, B and excess Al (which served as flux), were mixed with a composition of Y_0.56_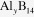 (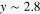). This mixture was pressed using cold isostatic pressing and heated around 1853 K for 8 h in vacuum (10 Pa). After heating, the sample was crushed and washed in NaOH to dissolve excess Al. Then the sample powder was consolidated by using spark plasma sintering at 1823 K for 5 min at 80 MPa.

XRD measurements were performed using an Ultima III system (Rigaku) with a Cu-K*α* radiation source. The Rietan-FP software package was used to characterize the samples [26]. TE power was measured with a TE measurement system (ZEM-2, ULVAC) by the four-probe method and the differential method.

For the first-principles calculation, we used the projected augmented wave method [27, 28] as implemented in the Vienna *Ab initio* Simulation Package [29]. Exchange–correlation energy was calculated within the generalized gradient approximation. In the present study, we used the functional proposed by Perdew and Wang [30]. Total energy was minimized over the degrees of freedom of both the electron density and the ionic positions by using the conjugate gradient iterative minimization technique. The cutoff energy for the plane wave expansion was taken to be 318.6 eV. Brillouin zone integration was performed using sets of 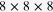 *k* points in all cases, which was sufficiently large to obtain close convergence.

**Figure 2:**
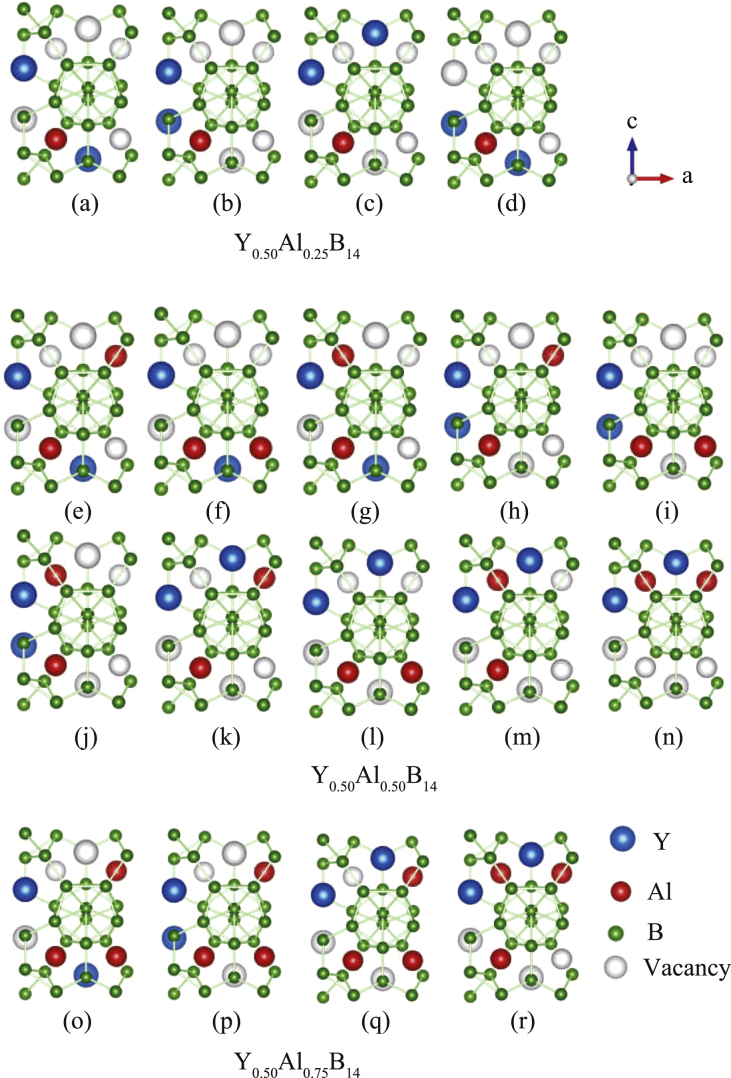
Possible configurations of Y, Al, and B in a single orthorhombic unit cell of YAlB_14_ with four formula units for (a)–(d) Y_0.50_Al_0.25_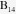, (e)–(n) Y_0.50_Al_0.50_B_14_, and (o)–(r) Y_0.50_Al_0.75_B_14_.

In order to examine the nonstoichiometric concentrations of Y and Al, we introduced model systems where the occupancies of Y and Al atoms were changed within one orthorhombic cell. Since YAlB_14_ compounds have four formula units per cell, we can consider compounds with *y* = 0.25, 0.50, and 0.75. In all the simulations, *x* was fixed at 0.50 since experimental studies have revealed that the Y site shows little variation. These compositions are sufficient to effectively analyze the dependence of the Seebeck coefficient on the concentrations of metal species. This is due to the experimentally observed change in Seebeck coefficient from positive to negative at around *y* = 0.5. In the present study, all possible configurations within an orthorhombic cell were calculated, and the electronic structures were analyzed for the energetically most stable ones (section 3). Figure 2 shows all possible configurations for Y_0.56_Al_*y*_B_14_ with (a)–(d) *y* = 0.25, (e)–(n) *y* = 0.50, and (o)–(r) *y* = 0.75, respectively. Here, blue, red, and green spheres represent Y, Al, and B atoms, respectively. For *y* = 0.25 and *y* = 0.75, there are four different configurations, and for *y* = 0.50, there are ten different configurations. Again, the structures illustrated in the figures are viewed directly above the *a–c* plane. It should be noted that these models enable us to analyze the effects of local atomic configurations and displacements from the ideal lattice points.

## Results and discussion

**Figure 3:**
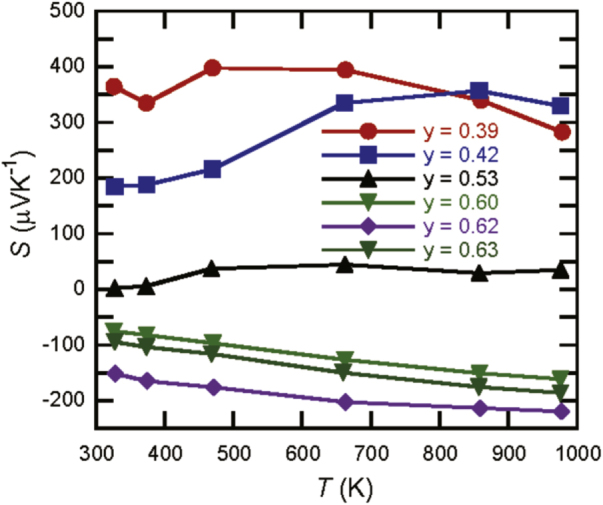
Experimentally obtained Seebeck coefficient values of 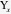 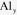 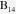. New data for y = 0.39 is plotted together with data from [22]. Lines are a visual guide.

First, we plot the experimentally obtained Seebeck coefficients of the Y_*x*_Al_*y*_B_14_ 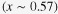 samples for different Al occupancies in [22] and for *y* = 0.39 in figure 3. Striking variation in the Seebeck coefficient ranging between large positive and negative values can be clearly observed. In the previous report [22], there was only one sample with p-type composition; here, however, variation was further confirmed for a larger range of compounds and the samples with lower *y* occupancy tended to show larger positive values of the Seebeck coefficient.

The temperature dependence of the *y* = 0.39 sample shows a difference with the higher aluminum concentration samples, with the Seebeck coefficient at relatively low temperatures exhibiting large values. At the limit of no aluminum, a related compound to Y_*x*_Al_*y*_B_14_ is known as an YB_25_-type compound with a composition of Y_*x*_B_14_ (x ∼ 0.55) [31] whose crystal structure is similar to Y_*x*_Al_*y*_B_14_ [32]. Recently sizable amounts of ‘YB_25_’ (Y_*x*_B_14_ (x ∼ 0.55)) were synthesized and their TE properties were measured. Similar to the more boron-rich and insulating compound YB_66_ [33], the Seebeck coefficients show large values of ∼1000 *μ*VK^−1^, around room temperature which decrease with temperature but still have values around 400 *μ*VK^−1^ at 1000 K [34]. The trend we observe for decreasing aluminum content *y* appears to approach this behavior. Incidentally, in terms of overall TE performance, both YB_66_ [33, 35] and ‘YB_25_’ [34] have small power factors despite the large Seebeck coefficient values because of the high electrical resistivity. This tendency is also observed for Y_*x*_Al_*y*_B_14_ with the TE power factor tending to be lower for the low aluminum concentration samples [22].

As regards a compromise between the TE properties, for the higher borides, it appears that a good compromise for the power factor is reached when the electrical conductivity attains relatively high values. For example, the aluminoboride with the highest power factor of 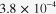 V^−2^ K^−2^ 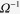 m^−2^, was achieved for the 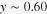 sample with resistivity below 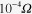 [22]. Incidentally, the power factors of the aluminoborides are still affected by the samples being not fully densified and there is room for improvement. The borosilicides RB_44_Si_2_ also have significantly higher power factors [36] than YB_66_ and ‘YB_25_’ in which the Seebeck coefficients are around 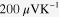 and electrical resistivity being below 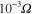 at high temperatures which is again relatively low for higher borides. In regard to the thermal conductivity, the aluminoborides [22, 34] are significantly higher than YB_66_ [4, 5] and RB_44_Si_22_ [6–8] and the difficulty in finding an optimum TE higher boride system still remains. However, in these initial stages of research, the figure of merit *ZT* is around 0.1 for both the n-type aluminoboride and the p-type RB_44_Si_2_ compound, so these are two promising systems to investigate further.

Next, we theoretically analyzed the structural properties of the samples by comparing with experimentally obtained values. First, table 1 shows the experimentally obtained structural parameters for six different concentrations of Y and Al. While table 2 shows the optimized structural parameters and relative total energy, which is defined as the total energy difference from the energetically most stable configuration per formula unit for each *x* and *y* obtained by first-principles calculations. The structures in figure 2(b), (h), and (p) are the energetically most stable configurations for Y_0.50_Al_0.25_B_14_ , 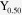
Al_0.50_B_14_ , and Y_0.50_Al_0.75_B_14_, respectively. For all of the most stable configurations, Y atoms occupy positions in the *b–c* plane, and the volume increases with increasing Al concentration.

**Table 1.** Experimentally obtained structural parameters of Y_*x*_Al_*y*_B_14_.

*x*	*y*	*a* axis (Å)	*b* axis (Å)	*c* axis (Å)
0.547(2)	0.394(4)	5.8458(8)	10.3401(14)	8.1766(11)
0.575(2)	0.411(3)	5.8118(6)	10.3518(10)	8.1889(8)
0.544(2)	0.532(4)	5.7816(7)	10.3409(12)	8.1444(9)
0.554(2)	0.601(4)	5.7947(7)	10.3757(12)	8.1609(10)
0.511(1)	0.620(3)	5.8182(3)	10.4105(5)	8.1901(5)
0.521(1)	0.634(2)	5.8162(3)	10.4082(7)	8.1886(5)

**Table 2.** Optimized structural parameters, and relative total energy per formula unit (fu). Here, the relative total energy is defined as the total energy difference from the energetically most stable configuration for *x* = 0.50 and each *y*. Indexing of the configuration is defined in figure 2.

	*y*	*a* axis (Å)	*b* axis (Å)	*c* axis (Å)	Relative total energy (eV/fu)
(a)	0.25	5.73	10.32	8.17	0.031
(b)	0.25	5.66	10.32	8.17	0.000
(c)	0.25	5.70	10.31	8.16	0.470
(d)	0.25	5.60	10.34	8.25	0.376
					
(e)	0.50	5.75	10.32	8.14	0.023
(f)	0.50	5.79	10.35	8.17	0.275
(g)	0.50	5.76	10.32	8.14	0.002
(h)	0.50	5.76	10.32	8.13	0.000
(i)	0.50	5.76	10.32	8.14	0.270
(j)	0.50	5.76	10.31	8.13	0.005
(k)	0.50	5.77	10.31	8.16	0.403
(l)	0.50	5.78	10.28	8.15	1.058
(m)	0.50	5.77	10.31	8.16	0.389
(n)	0.50	5.72	10.34	8.21	0.478
					
(o)	0.75	5.80	10.36	8.15	0.008
(p)	0.75	5.80	10.36	8.15	0.000
(q)	0.75	5.82	10.33	8.16	0.732
(r)	0.75	5.81	10.35	8.17	0.208

For most configurations, there are noncubic distortions in the optimized structures, but generally these are negligibly small (with values lower than 0.7%). The variation in the values of the total energy difference from the most stable configurations corresponding to a given *y* is rather small in most cases. For example, for Y_0.50_Al_0.25_B_14_ and Y_0.50_Al_0.75_B_14_, the energy differences between the most stable and the second most stable configurations are 0.031, and 0.008 eV per formula unit, and for Y_0.50_Al_0.50_B_14_, the energy difference between the most stable and the second or third most stable configurations are 0.002 or 0.005, respectively. While others have much higher energy and are unlikely to form. Therefore, these compounds are expected to have a random distribution of Al and Y atoms around room temperature with almost the same lower energy configurations for each composition, which would lead to orthorhombic lattice parameters. Comparing the calculated lattice constants for Y_0.50_Al_0.50_B_14_ (configuration in figure 2(h)) with the experimental values in table 1, the differences are less than 1%, although there is a small deviation in *x* and *y* among the results. This indicates that the calculations were highly accurate.

**Figure 4:**
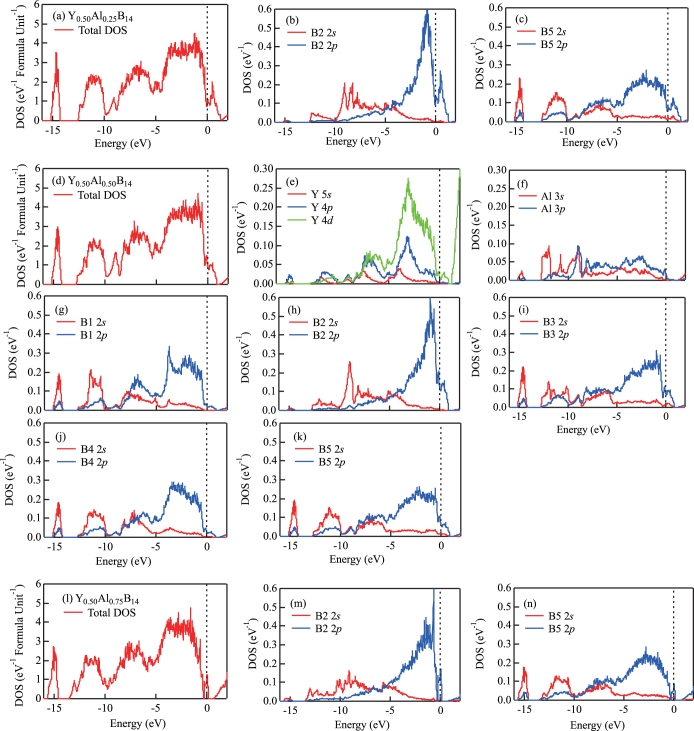
(a)–(c) Total density of states (DOS) and site-projected partial density of states (PDOS) at B2 and B5 for Y_0.50_Al_0.25_B_14_ with the atomic configuration of figure 2(b). (d)–(k) Total DOS and PDOS of Y, Al, B1, B2, B3, B4, and B5 for Y_0.50_Al_0.50_B_14_ with the atomic configuration of figure 2(h). (l)–(n) Total DOS and PDOS of B2 and B5 for Y_0.50_Al_0.75_B_14_ with the atomic configuration of figure 2(p). The Fermi level corresponds to an energy of 0 eV.

Next, we analyzed the variation in the electronic structure of the compounds due to Al doping. Figures 4(a)–(c) show total density of states (TDOS) and site-projected partial density of states (PDOS) at B2 and B5 for Y_0.50_Al_0.25_B_14_ with the atomic configuration of figure 2(b). Figures 4(d)–(k) show TDOS and PDOS of Y, Al, B1, B2, B3, B4, and B5 for Y_0.50_Al_0.50_B_14_ with the atomic configuration of figure 2(h). Furthermore, figures 4(l)–(n) show TDOS and PDOS of B2 and B5 for Y_0.50_Al_0.75_B_14_ with the atomic configuration of figure 2(p). The Fermi level is indicated with a dashed vertical line originating from 0 eV on the abscissa.

From figures 4(d)–(k), it is clear that the main contribution to the TDOS is due to 2*s* and 2*p* orbitals of B atoms that hybridize strongly at the nearest neighbor sites. The peak around -15 eV is mainly from 2*s* orbitals of boron, which has weak hybridization with Al 3*s* and 3*p* orbitals. Al atoms contribute only a small PDOS in the occupied region, and this implies charge transfer from Al atoms to the neighboring boron site, namely, B2. There are similar effects in the case of compounds with other concentrations of Al atom, and this behavior can be observed by comparing TDOS in figures 4(a), (d), and (l) corresponding to different concentrations of Al. Figures 4(b), (c), (h), (k), (m) and (n) show the change of the PDOS at B2 and their neighboring B5 at around Fermi level.

It is interesting to analyze the DOS behavior with respect to Al concentration. This is because the Seebeck coefficient is proportional to the first derivative of DOS, which concerns the energy around the Fermi level. That is,

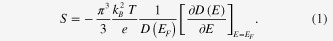
Here, *S* is the Seebeck coefficient, *D*(*E*) is the DOS, 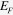 is the Fermi level, and *e* is the elementary charge. Considering the DOS immediately under the Fermi level in figures 4(a)–(c), we can see a shift in 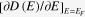 from negative through zero to positive values as the Al concentration increases. In the present study, the values, 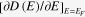, are estimated as –3.75, 0.30, and 1.01 eV^−2^ for Y_0.50_Al_0.25_B_14_, Y_0.50_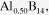 and Y _0.50_Al_0.75_B_14_, respectively. This is likely because doping of Al changes the shape of DOS, which is dominated by the 2*p* electrons of B. The 2*p* electrons of B hybridize with neighboring Al orbitals, and this effect controls the transport properties of the compound. This behavior suggests that the Seebeck coefficient can be controlled by changing the concentration and distribution of atoms in the lattice of yttrium aluminoborides.

To analyze how neighboring Al–B2 pairs change with Al concentration, table 3 shows the Al–B2 bond lengths and the corresponding number of bonds for Y_0.50_Al_*y*_B_14_. The indexing of the configuration in the table is defined in figure 2. Although the average bond length increases slightly with *y*, the number of bonds increases with *y*. Therefore, increasing Al concentration is expected to promote hybridization between Al and B2.

**Table 3.** Al–B2 bond lengths and the corresponding number of bonds, *n*, for Y_0.50_Al_*y*_B_14_. Indexing of the configuration is defined in figure 2.

	*y*	Length of Al–B2 bond ( 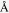 )	*n*
(b)	0.25	2.03	2
		2.05	2
			
(h)	0.50	2.06	8
			
(p)	0.75	2.03	2
		2.04	2
		2.05	2
		2.07	2
		2.11	4

**Figure 5:**
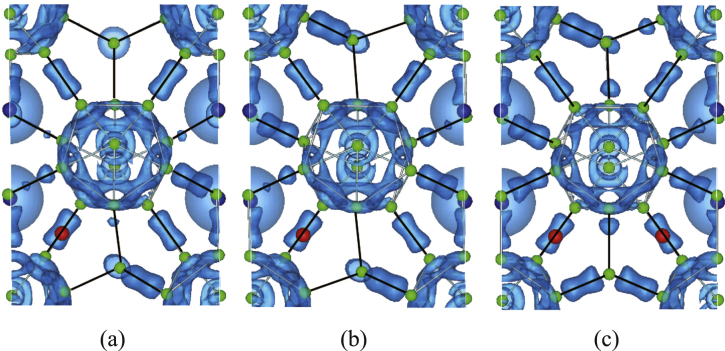
(a)–(c) Isosurface of 0.78 electron 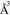 for the electronic charge density of Y_0.50_Al_0.25_B_14_, Y_0.50_Al_0.50_B_14_, and Y_0.50_Al_0.75_B_14_. Solid lines indicate the nearest neighbor inter-cluster bonds.

The change in hybridization of the compounds can be clearly seen by analyzing the electronic charge density. In figure 5(a)–(c) we show the charge density distribution for the configurations (b), (h), and (p) in figure 2, respectively. These correspond to the isosurface of 0.78 electron 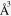. Solid lines indicate the nearest neighbor inter-cluster bonds. Here, blue, red, and green spheres represent Y, Al, and B atoms, respectively. It is noted that the Al atoms exist at the right upper site in figures 5(b) and (c). That is, they exist at the back side of the counter plot of the charge.

The overall total charge distributions for all cases are similar. That is, there is a high density of charge networks on B_12_ icosahedra and bridging B2 atoms. A strong covalent bonding can also be seen between B4 atoms that link neighboring icosahedra. B2 type bridging atoms form covalent bonds with neighboring B3 and B5 atoms. This is similar to the behavior of other systems such as Al*M*B_14_ systems with *M* = Li, Mg, and Na [37]. The strength of hybridization increases together with Al concentration. For example, this is clear when we consider inter-cluster bonds between B2 and B5 (see also figure 4). Furthermore, a small amount of charge also can be seen at around B3. This is because those boron sites are positioned near from Al sites compared with B1 and B4. That is, additional charge appears when Al concentration increases, which can be expected from the partial DOS shown in figure 4, and this effect contributes to the formation of the particular electronic properties.

Thus, the present simulation confirms the conclusion that the p–n characteristics of yttrium aluminoborides can be controlled by changing the Al concentration.

We note that particularly in the case of Al, there have been interesting TE effects for other completely different systems, such as Al-doping increasing the Seebeck coefficient for PbSe [38] and PbTe [39]. In the case studied here, we have demonstrated that the striking TE effect of Al was achieved by the unusual possibility in such metal borides to control the occupancy of the metal site, Al. If synthesis methods could be tried for other metal borides to enable such composition control for other metal species, it may lead to further excellent control of the TE properties.

It can be also noted that the contribution of the Al atoms to the DOS in the vicinity of the Fermi level is very small (figure 4). Coupled with the fact that the electronic density around the Al atoms (figure 5) does not appear to vary when the Al content increases, it is indicated that the Al atoms are weakly bonded. It can be imagined that this may be a similar situation to the rattlers in clathrates or skutterudites which contribute in significantly lowering the thermal conductivity [40, 41]. However, the thermal conductivity of borides appears to be quite complex, with different mechanisms like crystal complexity, disorder, and possible symmetry related effects contributing to the relative low thermal conductivity [7, 8], and actually the lattice thermal conductivity of aluminoborides is relatively high [34] compared to RB_44_Si_2_ [6–8] for example. In any case, the bonding of these interesting compounds should be investigated in detail in future works.

## Summary

Excellent control of p–n characteristics was recently reported for yttrium aluminoborides Y_*x*_Al_*y*_B_14_, which was found to be the result of the surprisingly free variation in the occupancy of Al sites. In the present work, this behavior was elucidated through theoretical investigations, namely first-principles calculations based on detailed XRD data. Additional samples were also experimentally prepared to illustrate the tendencies in p-type and n-type behavior observed when varying Al concentration.

The simulations revealed stable configuration of the atomic sites, indicating that such control of the occupancy of the metal sites was possible. A shift from positive through zero to negative values of the Seebeck coefficient was clearly demonstrated by determining the density of states for different concentrations of metal species.
